# Freeze-Dried Gellan Gum Gels as Vitamin Delivery Systems: Modelling the Effect of pH on Drying Kinetics and Vitamin Release Mechanisms

**DOI:** 10.3390/foods9030329

**Published:** 2020-03-11

**Authors:** Valentina Prosapio, Ian T. Norton, Estefania Lopez-Quiroga

**Affiliations:** School of Chemical and Engineering, University of Birmingham, Birmingham B15 2TT, UK; I.T.NORTON@bham.ac.uk (I.T.N.); E.Lopez-Quiroga@bham.ac.uk (E.L.-Q.)

**Keywords:** freeze-drying, gellan gum, modified pH, riboflavin, drying kinetics, release mechanism, model discrimination

## Abstract

Freeze-dried gellan gum gels present great potential as delivery systems for biocompounds, such as vitamins, in food products. Here, we investigate the effect of modifying the gel pH—prior to the encapsulation process—on drying and release kinetics, and on delivery mechanisms from the substrate. Gellan gum gels were prepared at pH 5.2, 4 and 2.5 and loaded with riboflavin before being freeze-dried. Release tests were then carried out at ambient temperature in water. Five drying kinetics models were fitted to freeze-drying experimental curves using regression analysis. The goodness-of-fit was evaluated according to (i) the root mean squared error (ii), adjusted R-square (iii), Akaike information criterion (iv) and Bayesian information criterion. The Wang and Singh model provided the most accurate descriptions for drying at acidified pH (i.e., pH 4 and pH 2.5), while the Page model described better freeze-drying at pH 5.2 (gellan gum’s natural pH). The effect of pH on the vitamin release mechanism was also determined using the Korsmeyer–Peppas model, with samples at pH 5.2 showing a typical Fickian behaviour, while acidified samples at pH 4 combined both Fickian and relaxation mechanisms. Overall, these results establish the basis for identifying the optimal conditions for biocompound delivery using freeze-dried gellan gels.

## 1. Introduction

Bioactive compounds used to enrich foods and beverages, such as vitamins, proteins or antioxidants, are highly sensitive to light, temperature and oxygen [1], undergoing degradation reactions (e.g., oxidation or pigment destruction) during processing that decrease their bioavailability [2]. To preserve them from degradation, those compounds can be encapsulated in suitable substrates according to the chosen functionalities [3] or required delivery rates; the release of a bioactive compound within the human body could be fast (mouth release) or prolonged over time (digestive tract release). The choice of the encapsulation technique is then key to preserving the biocompound and creating a suitable carrier microstructure—e.g., highly porous matrices can enhance mass transfer, leading to faster release rates. This makes freeze-drying a convenient technique for encapsulation of active biocompounds [4,5], as it helps with keeping the original porous structures of products, and its low temperature conditions also contribute to minimising degradation reactions [6,7].

One of the most versatile substrates employed in bioprocessing applications (i.e., food, pharma and healthcare technologies) is gellan gum gel. This is a non-toxic, biocompatible and biodegradable polymer [8] that has been extensively used as (i) a texturiser and gelling agent [9] in food applications; (ii) to formulate oral, nasal and ophthalmic formulations [10,11]; and (iii) as a scaffold for tissue regeneration [12,13]. A recent study [14] focused on the development of dried-gel structures from hydrocolloids has revealed the potential of gellan gum gels to be used as “controllable” carrier, showing that is possible to modulate the freeze dried-gel properties (i.e., target different microstructures and therefore different drying and rehydration kinetics) by modifying the pH of the initial gel solution.

To explore this promising path, this work focuses on the characterisation of freeze-dried gellan gum gels at different pHs as vitamin delivery systems. Freeze-drying kinetics, as well as release mechanisms and rates have been investigated using both empirical and modelling approaches. Gellan gum gels were prepared at different pHs (i.e., 5.2, 4 and 2.5) and then loaded with riboflavin (vitamin B2) before freeze-drying. Experimental drying curves were fitted to five common food drying models [15] (i.e., Newton, Page, Henderson and Pabis, logarithmic and Wang and Singh), and the effects of different pHs on freeze-drying kinetics were assessed. In addition, Information Theory criteria (Akaike and Bayesian information criteria) were used to discriminate the models [16] attending to their accuracy and complexity (i.e., number of parameters involved). The effect of pH on vitamin release (at ambient temperature) has been also studied using the classical Korsmeyer–Peppas model [17,18], and corresponding delivery mechanisms revealed. The findings of this work can help in the design of targeted freeze-dried gellan gum microstructures for the controlled delivery of active biocompounds in functional foods applications.

## 2. Materials and Methods

### 2.1. Materials

Low acyl (LA) gellan gum powder was provided by CPKelco (CPKelco, UK). Citric acid (purity 99%) and riboflavin (purity 98%) were supplied by Sigma-Aldrich (Sigma-Aldrich, Dorset, UK). All materials were used as received.

### 2.2. Preparation of Riboflavin-Loaded Gellan Gum Gels

Gellan gum powder was dissolved in distilled water at a concentration of 2% (*w/w*) and stirred at 85 °C for 2 h to ensure complete mixing [19]. This resulted in a gel solution at pH 5.2 (natural pH) (Seven compact Benchtop pH meter, Mettler Toledo UK). The gellan solutions at pH 4 and pH 2.5 were obtained by adding increasing quantities of citric acid at a 0.3 mol/L concentration [14]. All the solutions were placed in cylindrical moulds (diameter = 22 mm) for gelation [19]. Once the gels were formed, the moulds were cut into regular pieces (height = 15 mm,) and the resulting samples were soaked in a 2.7 × 10^−4^ mol/L riboflavin solution for 18 h. Finally, the loaded gels were washed with distilled water and blotted with paper to remove the vitamin settled on the surface.

### 2.3. Freeze Drying

The riboflavin-loaded gels were frozen at −20 °C for 24h and then freeze-dried at increasing processing times, from 2 h up to 18 h, in a bench top freeze dryer (SCANVAC Coolsafe™, model 110-4, Lynge, Denmark) with condenser temperature of −110 °C and chamber pressure of 10 Pa [14]. The experiments were performed in triplicates, and each batch of freeze-dried samples was weighted to measure the water content.

### 2.4. Water Activity Analysis

Water activity (a_w_) of wet and dried gels was measured using an AquaLab^®^ dew point water activity meter (model 4 TE, Decagon Devices Inc., Pullman, WA, USA). The temperature-controlled sample chamber was set to 25 °C [14,15]. All analyses were carried out in triplicate.

### 2.5. Vitamin Release Experiments

Vitamin delivery analyses were performed using a UV–Vis spectrophotometer (Orion Aquamate, Thermo Scientific, UK) at 444 nm. The loaded gels were placed in stirred (250 rpm) distilled water (200 mL) at room temperature. To measure vitamin release, aliquots of 3 mL were withdrawn from the release medium, analysed with the spectrophotometer and poured back into the release medium. The vitamin content in the release medium was then expressed as normalised vitamin release (NVR), a dimensionless quantity defined as:

(1) $$NVR=\frac{V\left(t\right)}{V_{total}}$$

All analyses were carried out in triplicate.

### 2.6. Drying Kinetics

The kinetics of moisture loss during freeze-drying of the loaded gellan gum gels were described by five empirical models commonly employed to characterise drying kinetics in foods [15,16]: Newton, Page, Henderson and Pabis, logarithmic and Wang and Singh. Table 1 lists them all alongside their expressions. 

To fit the models to the experimental freeze-drying curves, the (dimensionless) moisture ratios (MRs) of the samples were calculated first from the measured water content data as follows [15]:

(2) $$MR=\;\frac{X\left(t\right)-X_{eq}}{X_{0}-X_{eq}}$$

where *X(t)* is the moisture content on a dry basis for the different processing times (h), *X_0_* is the initial moisture content (*w/w* d.b.) and *X_eq_* is the equilibrium moisture content (*w/w* d.b). The equilibrium moisture content for the dehydrated gels was calculated using the GAB model with measured water activities and parameters for gellan gums presented in [9]:

(3) $$\frac{a_{w}}{X_{eq}}=0.165+14.3a_{w}-13.2\;a_{w}{}^{2}$$

For all the models in Table 2, the unknow parameters (parameters ***a_j_*** and *k_i_*, with ***j*** = 1,2 and *i* = 1, …, (6) were estimated using regression analysis. The error (e) between the experimental (*θ*) and predicted (i.e., fitted) MR values ($\bar{\theta }$) [15],

(4) $$J=\overset{N}{\underset{i}{\sum }}e_{i}{}^{2}=\overset{N}{\underset{i}{\sum }}{\left(\theta_{i}-{\bar{\theta }}_{i}\right)}^{2},$$

was minimised for all the *i* measurements that formed the experimental data set of size *N* using a nonlinear least squares method (implemented in Matlab with tolerance 10^−10^).

The goodness-of-fit of each fitted model was then assessed using three statistical measures that take into account the complexity (i.e., number of parameters, *p*) of each model [20]. These were:

- The adjusted *R^2^* [15]:
  (5) $$R_{adj}^{2}=1-\frac{N-1}{N-p}\left(1-R^{2}\right)$$
  where *R^2^* is the regression coefficient of determination.
- The corrected Akaike information criterion (*AICC*) [15,21]:
  (6) $$AICC=AIC+\frac{2p\left(p+1\right)}{N-p-1}$$
  where *AIC* is the Akaike information criterion [21,22].
- The Bayesian information criterion (*BIC*) [20]:
  (7) BIC=pln(N)−2 ln(L)
  where *L* is the maximum log-likelihood of the estimated model.

The goodness of the fit (or the likelihood) can be increased by adding more parameters to the model. However, this will increase complexity and might result in overfitting (i.e., more parameters than can be estimated with the available data), all which is penalized with higher AICC and BIC values [15,20]. Therefore, the model with best performance will be the one with higher $R_{adj}^{2}$ and lower *AICC* and *BIC* values [20].

### 2.7. Kinetics and Mechanisms of Vitamin Release

The Korsmeyer–Peppas model [17] has been used to describe release kinetics and identify delivery mechanisms. It is a semi-empirical power law that relates the fractional release of vitamin/drug to the release time [17,18]:

(8) $$\frac{M\left(t\right)}{M_{\infty }}=k_{rel}t^{n_{rel}}$$

where the M(t) and $M_{\infty }\;$ are the cumulative amounts of drug released at time *t* (measured in hours *h*) and infinite time, respectively; the constant *k_rel_* (in $h^{-n_{rel}}$ units) relates to the structure and geometry of the delivery matrix (in this case, the freeze-dried gels); and the dimensionless exponent *n_rel_* is the release mechanism indicator. For cylindrical substrates *n_rel_* ≤ 0.45 defines Fickian mechanisms, while anomalous/non-Fickian delivery is described by n_rel_ > 0.45 [17,18]. Experimental release curves were fitted to Equation (8) using a nonlinear least squares method [15], and parameters *k_rel_* and *n_rel_* were estimated within 95% confidence intervals.

## 3. Results and Discussion

### 3.1. Effect of pH on Moisture Losses during Freeze-Drying

**Figure 1:**
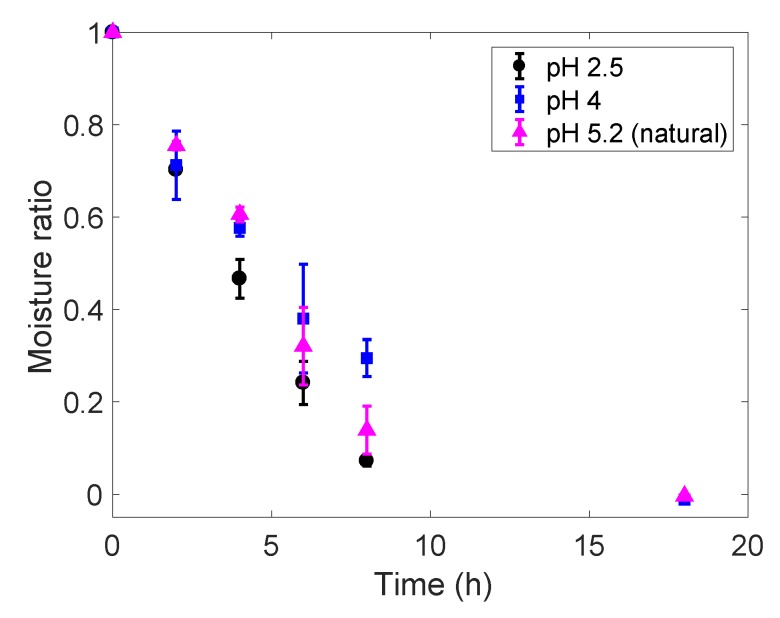
Moisture ratio evolution along time for 2% (*w/w*) gellam gums with pH 2.5 (black dots), pH 4 (blue squares) and pH 5.2 (magenta triangles) loaded with riboflavin during the conducted freeze-drying experiments.

Figure 1 shows a comparison of the drying curves, in terms of the moisture ratio (MR) and freeze-drying processing times (h), for gellan gum gels at pH 2.5, 4 and 5.2 (natural) loaded with riboflavin. Gels at pH 2.5 exhibit the fastest drying rates, with most of the moisture content (MR~0.25) removed during the first 6 h of the freeze-drying process. On the other hand, samples at pH 5.2 and 4 followed a very similar drying trend up to the first 4 h of processing. From this time onwards, the samples at pH 4 present a significantly slower drying rate; i.e., MR~0.3 at t = 8 h compared to MR~0.15 for pH 5.2 at the same time. The three samples were completely dried (i.e., free water totally removed) by the end of the freeze-drying experiments at t= 18 h, independent of their pHs.

The fastest drying rates observed for the gels at lower pHs can be explained by the effects of acidifying the gel solution. As reported by Cassanelli et al., [14], the acidification step both enhances ice crystal nucleation and weakens the gel structure at pH values as low as pH 2.5. The combination of these two effects might favour a more interconnected pore structure—i.e., more nuclei will lead to more crystals that will find lower resistance in the weak gel structure to form a network. This could lead to faster drying rates and also affect the strength of the rehydrated structure.

### 3.2. Freeze-Drying Kinetics: Parameter Estimation and Model Discrimination

Estimated parameters for all the drying models considered in this work (i.e., Newton, Page, Henderson and Pabis, logarithmic and Wang and Singh) are listed in Table 2, together with the RMSE (root mean square error) for each fitting and the results corresponding to the goodness-of-fit of each model. According to these results, the models that provide more accurate descriptions for the drying kinetics are the Page (Table Equation 1) and the Wang and Singh (Table Equation 5) models, both presenting $R_{adj}^{2}\sim 0.99$ (in average) for all pH values.

For samples at modified pHs (i.e., pH 2.5 and 4), the Wang and Singh model presents the lowest RMSE (3.52 × 10^−4^ for pH 2.5) and the highest $R_{adj}^{2}$, while for the freeze-dried gels at pH 5.2, the Page model is the model that presents the best fitting (RMSE = 0.040 and $R_{adj}^{2}=0.986$). This is in agreement with fittings reported in Cassanelli et al. [19], which showed the Page model as the best option to describe the freeze-drying kinetics of non-loaded gellan gels at natural pH.

**Figure 2:**
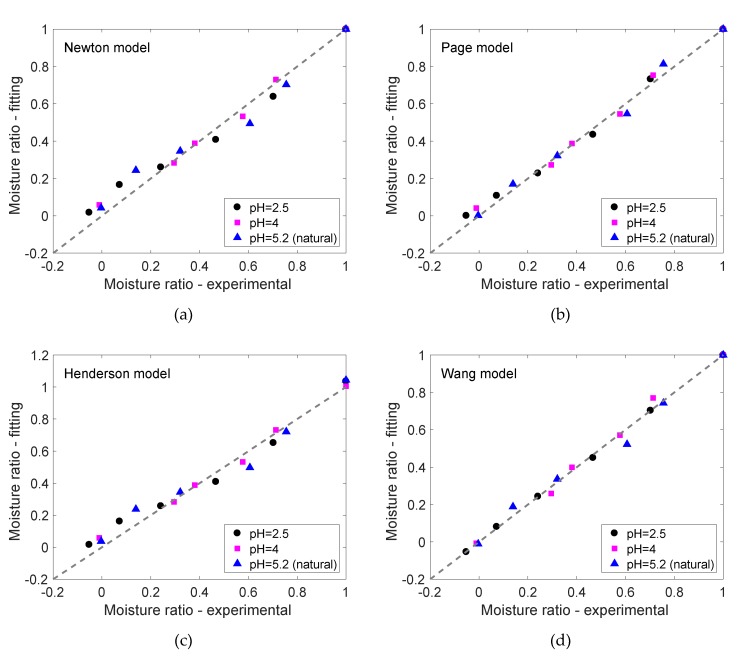
Correlation between predicted and experimental moisture contents for freeze-dried 2% (w/w) gellan gum samples for: (**a**) Newton model (Table Equation 1), (**b**) Page model (Table Equation 2), (**c**) Henderson and Pabis model (Table Equation 3) and (**d**) Wang model (Table Equation 5).

The goodness-of-fit for all models is illustrated in Figure 2, where experimental values are plotted against predicted moisture ratios for each drying model at all pH studied. This graph also shows the accuracy of the Page and Wang models, for which most of the predicted points lie on the correlation line (see Figure 2b,d).

When comparing models with similar accuracies, the *AICC* criterion constitutes the best measure to discriminate models, with more negative *AICC* values indicating better model performances. According to this, if the Newton and Page models were compared at pH 4—the pH at which both models show very similar RMSE,$\;R_{adj}^{2}$ and *BIC*—the lower AICC (−21.39) of the Newton model would make it the preferred one. This criterion is also an indicator of the complexity (e.g., number of parameters) of the assessed models—the Newton model involves a single parameter (*k_1_*), compared to the two needed in the Page model (*k_2_, n*). On the other hand, the logarithmic model (Table Equation 4), with the highest number of parameters considered (*p* = 3), presents the less negative *AICC* values at each pH.

#### Effect of pH on the Drying Kinetic Parameters

The effect of pH on drying kinetic parameters has been determined by analysing the values of the constants in Newton (Table Equation 1) and Henderson and Pabis (Table Equation 3) models. These two models are derived from Newton’s cooling law and Fick’s Second law [16], respectively, so their constants enclose physical meaning—as opposite to the Page and Wang and Singh models that are purely empirical [16].

Parameters *k_1_* (Newton) and *k_3_* (Henderson) in Table 2, both time constants (*h^−1^*), characterise the drying rates of the system, while *a_1_* (Henderson) is a dimensionless parameter related to the shape and structural properties of the samples [16].

For gellan gum gels at different pHs, both Newton and Henderson rate parameters (i.e., *k_1_* and *k_3_*, respectively) show very similar trends. The higher values (*k_1_* = 0.225 h^−1^ and *k_3_* = 0.232 h^−1^) correspond to samples at pH 2.5, indicating a faster dehydration process. On the other hand, rate constants for samples at pH 4 are the lowest (*k_1_* = 0.157 h^−1^ and *k_3_* = 0.158 h^−1^), which relates to the slower drying rate of these samples. This is in agreement with differences on moisture ratios (MR) at different pHs discussed in Section 3.1.

The values of constant *a_1_* are again similar for samples at pH 2.5 and 5.2 (*a_1_* = 1.036 and *a_1_* = 1.043), which suggests no significant structural differences at those pH values. However, the value of *a_1_* for freeze-dried gels at pH 4 (*a_1_* = 1.006) suggests changes in microstructure that might be behind the different drying rates observed at this particular pH. These findings are in agreement with the mechanical properties (i.e., higher gel strength and Young’s modulus) reported in Cassanelli et al. [14] for gellan gels at pH 4 before and after freeze-drying—“stronger” gels might make ice nucleation and growth difficult, and therefore affect the freeze-dried microstructures of the gels.

### 3.3. Riboflavin Release from Freeze-Dried Gellan Gels at Different pHs

**Figure 3:**
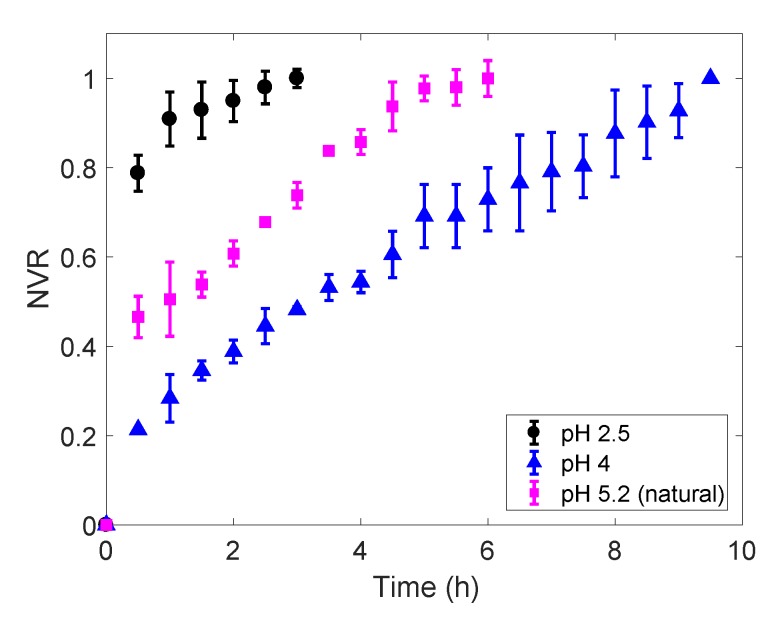
Release curves for the riboflavin encapsulated in freeze-dried 2% (*w/w*) gellan gums with different pHs. The vitamin content in the release medium is expressed as normalised vitamin released (NVR). Error bars correspond to triplicate tests.

Figure 3 presents experimental riboflavin release curves from freeze-dried gels prepared at different pHs, plotted as normalised vitamin released (NVR) across time. Data in this graph show significant differences in release times: freeze-dried gels at pH 4 completed the vitamin release in approximately 9.5 h; gels at natural pH (5.2) were fully unloaded after 6h, and total vitamin delivery took 3h for gels at pH 2.5. Samples at pH 2.5 presented a weak structure—in accordance with strength at fresh and freezing stages—that lead to breakage during the release experiments. This increased the surface area of the gels in contact with the release medium, which explains the shorter delivery times.

The observed differences in the riboflavin release times to the medium can be related to the different microstructures and mechanical properties of the gels. Both Norton et al. [23] and Cassanelli et al. [14] reported that freeze-dried gellan gum gels at pH 4 exhibited an aggregated and rigid structure. This can impede mass transfer within the gel, increasing the time needed to release the vitamin completely from the substrate and leading to longer delivery processes. A much lower level of aggregation and very weak structures were reported for freeze-dried gels at pH 2.5 [14,23], which is also in agreement with our experimental observations. According to the same authors, unloaded freeze-dried gels at natural pH (pH 5.2) exhibit intermediate levels of aggregation [14,24], explaining the also intermediate release times shown in Figure 3.

### 3.4. Delivery Mechanisms at Different pHs

To estimate the value of the dimensionless parameter *n_rel_* in Equation (8), and therefore determine the release mechanism governing riboflavin delivery, the portion of the release curves (Figure 3) corresponding to the first 60% of the total released vitamin—i.e., release curve portions such that $\frac{M\left(t\right)}{M_{\infty }}=NRV\leq 0.60$—were fitted to the Korsmeyer–Peppas model [17,18]. Samples at pH 2.5 were not considered in this analysis, as they broke into several pieces during the release tests, leading to delivery conditions out of the scope of this work. Table 3 lists the parameters *k_rel_* and *n_rel_* (95% CI) estimated at pH 5.2 and pH 4 (see Table 3 for parameter units). These results are discussed next.

#### 3.4.1. Release from Freeze-Dried Gellan Gels at pH 5.2

Riboflavin delivery from gels at natural pH (pH 5.2) is characterised by a shape constant *k_rel_* = 0.509 diffusional coefficient *n_rel_* = 0.131 (see Table 3 for the corresponding confidence intervals). According to the classification given in [21,22], this indicates that the governing release mechanism is purely Fickian, as *n_rel_* = 0.131 < 0.45, which is the limiting value of the diffusional coefficient for Fickian transport mechanisms. Therefore, we can define an apparent diffusion coefficient *D_app_* (m^2^/s) for samples at pH 5.2 using a short-time approximation of Fick’s Second law [18]:

(9) M(t)M∞=4[Dapptπa2]12

**Figure 4:**
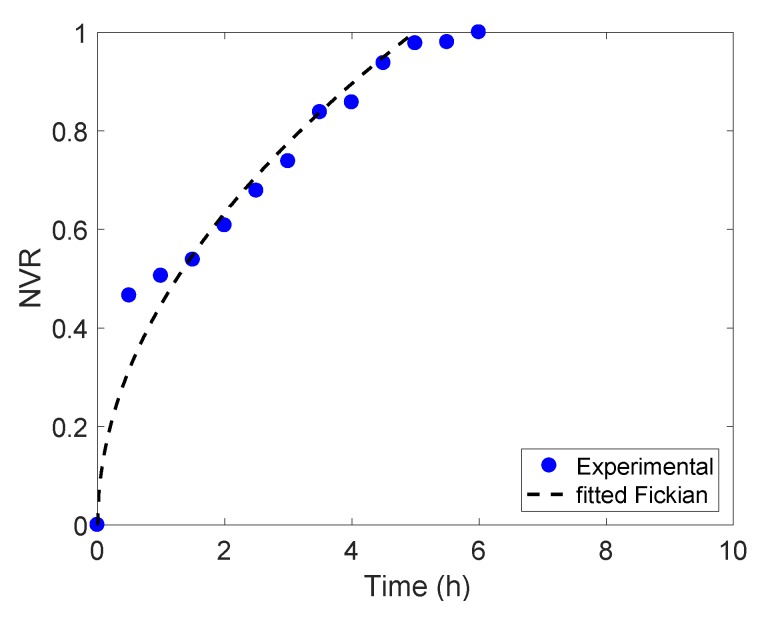
Predicted release curve for encapsulated riboflavin at pH 5.2 using estimated *D_app_* (dash --) compared to experimental curve (blue dots).

As the aspect ratio of the cylindrical gels is approximately in the order of 1, the predictive capabilities of the short-time solution include up to the 85% of the total vitamin release [22]. Thus, values such that $\frac{M\left(t\right)}{M_{\infty }}=NRV\leq 0.85$ in the release curve at pH 5.2 were fitted to Equation (9). This gave an estimated *D_app_* = 1.325 × 10^−9^ m^2^/s with 95% CI defined by [1.086 × 10^−9^, 1.564 × 10^−9^] m^2^/s. Figure 4 presents the comparison between the experimental and predicted release curve using *D_app_*, showing a good agreement between the modelled Fickian delivery mechanism and the experimental data—and thus confirming Fickian transport for riboflavin released from freeze-dried gellan gels at pH 5.2

#### 3.4.2. Release from Freeze-Dried Gellan Gels at pH 4

As shown in Table 3, the estimated shape constant and diffusional exponent at pH 4 were *k_rel_* = 0.287 and *n_rel_* = 0.472, respectively. These estimates (i) confirm the structural difference of samples at pH 4 discussed in previous subsections, as the value of *k_rel_* at pH 4 is almost half of the corresponding to pH 5.2, and (ii) indicate an anomalous delivery mechanism from freeze-dried gellan matrices at pH 4, since we estimated *n_rel_* > 0.45.

Anomalous mass transport can be defined as a mix between Fickian and non-Fickian mechanisms, for which the general form of the Korsmeyer–Peppas model presented in Equation (8) can be split into two contributions [24]:

(10) $$\frac{M\left(t\right)}{M_{\infty }}=k_{rel}t^{n_{rel}}=k_{1}^{rel}t^{m_{rel}}+k_{2}^{rel}t^{2m_{rel}}.$$

The first one ($k_{1}^{rel}t^{m}$) represents the Fickian part, while the second term ($k_{2}^{rel}t^{2m}$) accounts for the relaxational contribution [24], which is related to stresses and state transitions of polymeric matrices. The dimensionless diffusional exponent *m_rel_* in Equation (10) can be determined from aspect ratio (i.e., height/diameter of the sample) correlations. For the ratio characterising the cylindrical samples used here (~1.5), *m_rel_* = 0.43 [24].

Estimated values for both $k_{1}^{rel}=\;$0.264 (0.240, 0.289) and $k_{2}^{rel}$ = 0.022 (0.007, 0.038) were then obtained by fitting Equation (10) to the experimental release curves at pH 4 for NMC <0.60 with 95% confidence intervals (values in the parenthesis). Units for $k_{1}^{rel}$ and $k_{2}^{rel}$ are ($h^{-m_{rel}})$. These estimates give an idea of the relevance of each contribution. For samples at pH 4, $k_{1}^{rel}\gg k_{2}^{rel}$, showing that the release of riboflavin from the freeze-dried gels at pH 4 is mostly Fickian. This is confirmed by the vitamin release percentages due to each contribution calculated as Peppas and Sahlin [24]:

(11) $$P_{Fickian}=\frac{1}{1+\frac{k_{2}^{rel}}{k_{1}^{rel}}t^{m_{rel}}}\;;\;P_{non-Fick}=100-P_{Fickian}=P_{Fickian}\frac{k_{2}^{rel}}{k_{1}^{rel}}t^{m_{rel}}\;$$

**Figure 5:**
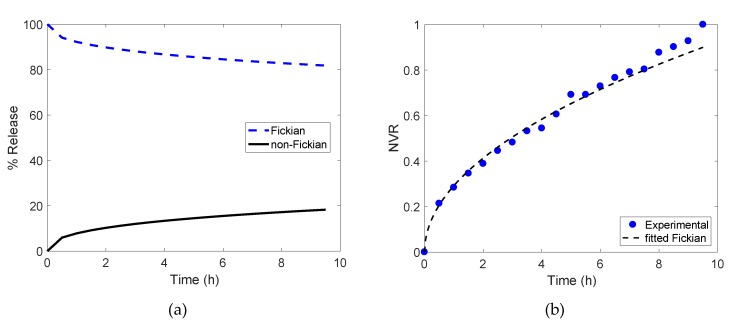
(**a**) Fickian and non-Fickian release percentages for riboflavin corresponding to sample with pH 4 when anomalous transport mechanism was considered. (**b**) Predicted release curve for encapsulated riboflavin at pH 4 considering pure Fickian mechanism and estimated *D_app_* (dash --) compared to experimental curve (blue dots).

They are presented in Figure 5a. Overall, the Fickian contribution is predominant along the release process, i.e., overall $P_{Fickian}>80\%$, with values closer to 90% at the initial times of the delivery test, while relaxation effects are more important towards the end of the experiment, as the delivery of riboflavin is closer to completion.

Given the relevance of Fickian transport during the release process at pH 4, and with the estimated diffusional exponent *n_rel_* so close to the Fickian limiting value of 0.45—confidence intervals for this parameter are actually cross-boarding this limit, i.e., (0.441, 0.504) as shown in Table 3—a hypothetical pure Fickian riboflavin delivery at pH 4 has been also assessed. Following the procedure explained in Section 3.4.1, an apparent diffusion coefficient D_app_ = 5.626 × 10^−10^ m^2^/s was estimated within a 95% confidence interval (5.409 × 10^−10^, 5.842 × 10^−10^) m^2^/s. This estimate together with the short time approximately described in Equation (9) was used to obtain a predicted release curve, which is presented in Figure 5b alongside the experimentally obtained curve. As the comparison reveals, the hypothetical pure Fickian mechanism describes the behaviour observed during the release tests well, and it could be used to predict riboflavin delivery—neglecting relaxation effects—from freeze-dried gellan gels at pH 4 with high accuracy.

## 4. Conclusions

This work demonstrates the potential to control biocompound release from freeze-dried gellan gum gels by modifying the pH of the substrate during gel formation, and prior to the encapsulation stage. As an exemplar of a relevant biocompound, riboflavin (i.e., vitamin B_2_) was used.

Freeze-drying kinetics, as well as release mechanisms, were experimentally investigated and modelled. Five different drying kinetics models were discriminated by accuracy and goodness-of-fit using statistical measures (i.e., RMSE, $R_{adj}^{2}$, *AICC* and *BIC*). For samples at natural pH (pH 5.2), the Page model provided the most accurate description of freeze-drying kinetics, while the Wang and Singh model predicted more accurately, the kinetics at acidified pH (i.e., 4 and 2.5).

Results revealed consistent differences in the behaviour of substrates at pH 4. Such differences reflect slower drying and release kinetics, as well as a different delivery mechanism—samples at natural pH (pH 5.2) exhibited Fickian transport, while acidified samples (pH 4) were characterised by an anomalous release mechanism, but with a predominantly Fickian contribution (80–90%).

Overall, this work shows the potential of modified pH freeze-dried gellan gum gel matrices for controlled riboflavin release, demonstrating that:

(i) These hydrogels could be used in different enriched food and/or beverage products.
(ii) Model-based approaches like the one presented here represent useful tools for the design of novel food formulations.

## Figures and Tables

**Table 1:** Drying kinetics models considered in this work to describe moisture loss during freeze-drying of riboflavin-loaded gellan gum gels with different pHs.

Drying model	Expression [15,16]	
Newton	$MR=e^{-k_{1}t}$	Table Equation (1)
Page	$MR=e^{-k_{2}t^{n}}$	Table Equation (2)
Henderson and Pabis	$MR=a_{1}e^{-k_{3}t}$	Table Equation (3)
Logarithmic	$MR=a_{2}e^{-k_{4}t}+b_{1}$	Table Equation (4)
Wang and Singh	$MR=1+k_{5}t+k_{6}t^{2}$	Table Equation (5)

Parameter units: (*h^−1^*) for *k_1_*, *k_3_*, *k_4_*, *k_5_*; (*h^-n^*) for *k_2_;* (*h^−2^*) for *k_6_; a_1_, a_2_* and *b_1_* are dimensionless.

**Table 2:** Regression and goodness-of-fit results for the drying kinetics models.

Model	Parameters	RMSE	R^2^_adj_	BIC	AICC
Newton					
pH 2.5	*k_1_* = 0.225	0.066	0.973	−13.71	−14.92
pH 4	*k_1_* = 0.157	0.038	0.988	−20.18	−21.39
pH 5.2	*k_1_* = 0.176	0.076	0.961	−11.94	−13.15
Page					
pH 2.5	*k_2_* = 0.117; *n* = 1.417	0.035	0.990	−20.61	−16.20
pH 4	*k_2_* = 0.132; *n* = 1.101	0.035	0.988	−20.76	−16.34
pH 5.2	*k_2_* = 0.071; *n* = 1.551	0.04	0.986	−19.10	−14.69
Henderson					
pH 2.5	*a_1_* = 1.036; *k_3_* = 0.232	0.064	0.968	−13.57	−9.15
pH 4	*a_1_* = 1.006; *k_3_* = 0.158	0.038	0.985	−19.63	−15.21
pH 5.2	*a_1_* = 1.043; *k_3_* = 0.185	0.073	0.955	−11.87	−7.45
Logarithmic					
pH 2.5	*a_2_* = 1.146; *k_4_* = 0.186; *b_2_ = −0.121*	0.042	0.981	−16.69	−4.07
pH 4	*a_2_* = 1.114; *k_4_* = 0.126; *b_2_ = −0.125*	0.021	0.994	−24.95	−12.32
pH 5.2	*a_2_* = 1.142; *k_4_* = 0.153; *b_2_ = −0.109*	0.064	0.954	0.89	−11.73
Wang and Singh					
pH 2.5	*k_7_* = −0.160; *k_8_* = 0.0056	3.52 × 10^−4^	0.999	−37.85	−33.44
pH 4	*k_7_* = −0.122; *k_8_* = 0.0037	0.005	0.990	−17.37	−21.78
pH 5.2	*k_7_* = −0.138; *k_8_* = 0.0045	0.045	0.983	−13.33	−17.75

Parameter units: (*h^−1^*) for *k_1_*, *k_3_*, *k_4_*, *k_5_*; (*h^-n^*) for *k_2_;* (*h^−2^*) for *k_6_; a_1_, a_2_* and *b_1_* are dimensionless.

**Table 3:** Fitted parameters (95% CI) for the Korsmeyer–Peppas release model and release mechanisms found.

	*k_rel_*	*n_rel_*	Release Mechanism
pH 2.5	-	-	-
pH 4	0.287 (0.277, 0.297)	0.472 (0.441, 0.504)	Anomalous transport
pH 5.2 (natural)	0.509 (0.502, 0.515)	0.131 (0.102, 0.161)	Fickian diffusion

Parameter units: ($h^{-n_{rel}}$)*; n_rel_* is dimensionless.
